# Dual-temporal inflow–outflow dependency modeling for short-term metro outflow prediction

**DOI:** 10.1371/journal.pone.0347131

**Published:** 2026-04-21

**Authors:** Wangxin Hu, Zhongxiang Huang, Jianrong Cai, Xiufang Zhao

**Affiliations:** 1 School of Transportation, Changsha University of Science and Technology, Changsha, China; 2 School of Civil Engineering, Hunan City University, Yiyang, China; National University of Defense Technology, CHINA

## Abstract

Recent advances in deep learning have substantially improved short-term metro passenger flow prediction. However, existing approaches often inadequately model the dependency of outflow on inflow and typically rely on predefined station correlation graphs, which limits modeling flexibility and representational capacity. To address these issues, this study decomposes the influence of inflow on outflow into short-term and long-term temporal components and proposes a dual-temporal inflow–outflow dependency model (DTIOD). DTIOD adopts an asymmetric feature extraction scheme to encode inflow and outflow sequences according to their distinct roles in forecasting. Instead of using predefined station correlation graphs or explicit spatial modules, the model employs a dual-branch cross-attention mechanism to capture inflow–outflow dependencies across multiple temporal scales, thereby enabling implicit learning of spatial correlations. In addition, sample-level origin–destination (OD) matrices are incorporated as additive attention biases to embed prior inter-station relationships and guide attention allocation. The outflow features are adaptively fused with the long-term and short-term inflow effect representations through learnable weights, and final predictions are generated by a fully connected layer. Experiments on the Hangzhou metro dataset show that DTIOD reduces RMSE (root mean squared error), MAE (mean absolute error), and WMAPE (weighted mean absolute percentage error) by 10.75%, 11.60%, and 6.84%, respectively, compared with the strongest baseline, while completing training within 70 seconds. These results demonstrate that DTIOD achieves a favorable balance between predictive accuracy and computational efficiency, indicating its practical applicability.

## 1 Introduction

Accurate short-term passenger flow forecasting is essential for improving metro operational efficiency, supporting refined passenger flow guidance, and responding promptly to emergencies. However, metro passenger flows are influenced by diverse factors such as land use patterns, meteorological conditions, public activities, and holiday effects, exhibiting significant nonlinearity and instability. As a result, achieving reliable short-term metro flow prediction remains a major challenge in intelligent transportation research.

Metro passenger flow forecasting has progressed from traditional statistical models to machine learning techniques, and more recently to deep learning-based frameworks. Statistical approaches such as Kalman filters [1] and autoregressive integrated moving average [2] capture basic temporal dependencies but exhibit limited capacity in modeling nonlinear patterns and complex disturbances. Machine learning methods, including decision trees [3], bayesian models [4], and support vector machines [5], improve nonlinear representation but rely heavily on manual feature engineering. As data availability and computational capacity continue to increase, deep learning methods have shown clear advantages in complex signal modeling, wireless sensing, and high dimensional pattern recognition because of their strong nonlinear representation capability [6–8]. Consequently, deep learning has become a dominant paradigm in metro passenger flow forecasting research [9]. Among these approaches, spatiotemporal fusion models [10] have been widely adopted as a central framework in both theoretical studies and practical applications. Building on this foundation, recent research has mainly explored three categories of mechanisms to further improve predictive performance.

The first category of mechanisms targets the restructuring and enrichment of spatiotemporal inputs to forecasting models. Temporal preprocessing strategies such as series decomposition [11] and reorganization [12,13] are commonly adopted to regulate nonstationary, nonlinear, and multi-scale dynamics in passenger flow sequences, offering temporally more tractable representations. In the spatial domain, interstation dependencies are encoded through the construction and refinement of station correlation graphs [14], including passenger flow similarity graphs [15], origin—destination (OD) flow graphs [16–18], point of interest (POI) similarity graphs [16,19], dynamic graphs [20–22], and other multi-relational graphs [23,24]. In addition, auxiliary variables such as calendar attributes, meteorological conditions, and operational schedules are often incorporated to model exogenous influences on passenger flow evolution, further improving predictive accuracy and generalization [25].

The second category of mechanisms focuses on improving spatiotemporal feature representation and interaction modeling through the refinement of temporal learning modules, spatial learning modules, and their integration strategies. In temporal modeling, convolutional operators and attention mechanisms are widely adopted to capture long-term trends and short-term dynamics in passenger flow [26–28]. For spatial feature learning, graph convolutional networks (GCNs) remain the dominant modeling paradigm. Variants such as the personalized enhanced GCN improve model responsiveness under peak-flow conditions and alleviate the noise sensitivity of conventional GCNs [29]. Moreover, recent studies increasingly emphasize coordinated spatiotemporal learning with multi-scale coupling, enabling temporal dependencies and spatial structures to be learned jointly and resulting in more robust predictive performance [30,31].

The third category of methods is grounded in fundamental metro travel behavior and explicitly models spatiotemporal dependencies between inflow and outflow. In metro systems, outflow at a station originates from earlier inflow at other stations, establishing a clear temporal order in which entry precedes exit and resulting in cross-station spatial coupling. Consequently, treating these flows as independent multi-variate inputs fails to capture their intrinsic dependency structures [11,24,31,32]. To address this limitation, several studies incorporate passenger flow formation mechanisms that characterize the propagation pathways and dynamic evolution of passenger movements from inflow to outflow, thereby enhancing interpretability while improving forecasting accuracy [16,17,33,34].

Overall, existing studies have substantially advanced metro passenger flow forecasting through input enhancement, structural optimization, and mechanism-driven modeling. Nevertheless, several critical limitations remain, which can be summarized as follows.

(1) Excessive reliance on predefined station correlation graphs. Most approaches depend on such graphs to model spatial relationships in passenger flows. However, simple adjacency graphs struggle to capture the multi-dimensional latent connections among stations, whereas overly dense graph structures often introduce redundant information that degrades performance. Although multi-graph and dynamic graph formulations partially alleviate these issues, their performance improvements are typically achieved at the cost of substantially increased model complexity. Moreover, graph construction is highly sensitive to data incompleteness and noise, which undermines the robustness of spatial relationship learning. To date, relatively few studies have investigated latent interstation passenger flow interactions without relying on predefined correlation graphs [35].
(2) Insufficient characterization of inflow—outflow relationships. The relationship between inflow and outflow in metro systems exhibits both short-term dynamics and long-term evolutionary patterns. Short-term dependencies arise from the passenger travel process. During a typical journey, passengers pass through entry, travel, and exit stages; therefore, outflow at a given time step is partly influenced by inflow in preceding time steps. In addition to these short-term effects, inflow and outflow also display pronounced long-term patterns. Commuting behavior illustrates this phenomenon. Passengers often exit near their workplaces during the morning peak and later enter the same stations during the evening peak to return home. In contrast, stations located in residential areas exhibit the opposite inflow and outflow pattern. Such regular travel behavior creates a relatively stable correspondence between inflow and outflow at the same station over longer time horizons and produces persistent long-term dependencies. However, most existing studies rely on OD data to characterize passenger flow relationships [16,17,33,34]. These data are derived from complete travel records and describe passenger movements between origins and destinations within short time intervals. Consequently, they are inherently limited to modeling short-term transformation patterns between stations.
(3) Existing strategies for exploiting OD data struggle to balance dynamic representational capacity with computational efficiency. One line of research constructs static OD correlation graphs from OD statistics aggregated at the dataset level [16–18]. While these graphs capture overall movement patterns, they fail to reflect the dynamic evolution of passenger flows. Another line constructs OD graphs at each time step to model instantaneous spatial distributions [13,22,33,34], at the expense of substantially increased computational cost. Consequently, achieving a principled trade-off between temporal responsiveness and computational tractability remains an open and practically significant challenge.

To address these limitations, this study examines the evolutionary patterns of inflow and outflow and decomposes the influence of inflow into short-term and long-term temporal components. On this basis, a dual-temporal inflow–outflow dependency model (DTIOD) is developed for metro outflow prediction. Since the dependency between inflow and outflow varies across temporal scales, the model aims to capture the multi-scale influence of historical inflow on future outflow. Attention mechanisms have recently been widely adopted for multi-scale temporal modeling and cross-modal feature integration because they enable dynamic weighting to capture complex dependencies [36–39]. Building on this capability, DTIOD employs a two-branch multi-head attention architecture within a unified embedding space to model both long-term and short-term dependencies from historical inflow to future outflow. A sample-level OD flow matrix is further incorporated as a learnable bias in the short-term branch, improving sensitivity to dynamic flow patterns while maintaining a lightweight model structure.

The main contributions of this paper are summarized as follows.

(1) A cross-sequence dual-temporal modeling framework with a dual-branch attention mechanism is proposed. Unlike existing approaches that primarily rely on OD data to characterize short-term passenger interactions among stations, the proposed framework explicitly models the evolutionary relationships between inflow and outflow across long-term and short-term temporal scales. This design enables the outflow representation of each station to selectively aggregate multi-scale inflow information from the entire network.
(2) In contrast to conventional spatiotemporal fusion approaches, DTIOD directly learns multi-scale dependencies between inflow and outflow without relying on predefined station correlation graphs or explicit spatial modules. A sample-level OD bias is incorporated into the attention computation to encode prior passenger flow relationships among stations. This design enables the model to capture short-term flow variations while maintaining computational efficiency.
(3) Experiments on real-world datasets demonstrate that DTIOD outperforms mainstream approaches in both predictive accuracy and training efficiency. Because the model relies solely on inflow and outflow data, it requires substantially lower data completeness than typical spatiotemporal fusion models, which improves its practical applicability for metro passenger flow forecasting.

The remainder of the paper is organized as follows. Section 2 describes the DTIOD architecture and its core modules. Section 3 presents the datasets and model configurations. Section 4 reports the experimental results and provides comparative analyses. Section 5 concludes the paper and discusses future research directions.

## 2 Problem description and model construction

### 2.1 Problem description

This study is motivated by travel behavior patterns that drive passenger flow dynamics across both short-term and long-term temporal scales. Accordingly, we decompose the influence of inflow on outflow into short-term and long-term components and explicitly model the resulting dual-scale temporal dependencies to enable accurate prediction of metro outflow in a future time interval. To formalize the problem setting, five key concepts are defined below.

Definition 1 (inflow and outflow time series).

Let $X^{in}$ and $X^{out}$ denote the inflow and outflow time series extracted from Automatic Fare Collection (AFC) data. The outflow time series $X^{out}$ is defined as


$$X^{out}=\left(\overset{out}{\underset{\text{0}}{x}},\overset{out}{\underset{\text{1}}{x}},\cdots ,\overset{out}{\underset{t}{x}},\cdots ,\overset{out}{\underset{T-1}{x}}\right)\in {\mathbb{R}}^{N\times T},$$ (1)

where N denotes the number of stations, and T is the total number of time steps after aggregating the data at a specified time granularity. The outflow vector at time step t, denoted as $\overset{out}{\underset{t}{x}}$, is defined as:


$$\overset{out}{\underset{t}{x}}=\left[\overset{out}{\underset{t,0}{x}},\cdots ,\overset{out}{\underset{t,n}{x}},\cdots ,\overset{out}{\underset{t,N-1}{x}}\right]\in {\mathbb{R}}^{N\times 1},$$ (2)

where $\overset{out}{\underset{t,n}{x}}$ represents the outflow volume at station n during time step t.

The inflow time series $X^{in}$, the inflow vector $\overset{in}{\underset{t}{x}}$, and the element $\overset{in}{\underset{t,n}{x}}$ are defined similarly.

Definition 2 (time-step-based OD tensor).

Based on AFC records with precise entry and exit timestamps, a time-step OD matrix $O_{t}\in {\mathbb{R}}^{N\times N}$ is constructed for each time step t. It is formed by identifying all passenger trips that terminate within t and retrieving their corresponding entry stations from AFC trajectories. The resulting matrix characterizes the distribution of passengers completing their trips during t, irrespective of their entry times. Compared with OD matrices constructed from the entire dataset or training set, the time‑step‑based OD matrix preserves finer‑grained spatiotemporal information. Since it is derived solely from trips ending at the current time step, it avoids the future information leakage that can occur when generating prediction samples from entry‑based OD matrices. Stacking these matrices for each time step forms a time‑step‑based OD tensor, formally defined as follows.


$$𝒪=\left(O_{\text{0}},O_{\text{1}},\cdots ,O_{t},\cdots ,O_{T-1}\right)\in {\mathbb{R}}^{T\times N\times N}.$$ (3)

Definition 3 (sample).

A complete sample for the DTIOD model comprises an outflow sequence, a long-term inflow sequence, a short-term inflow sequence, and a corresponding OD matrix. These components are extracted from the original outflow and inflow time series using sliding windows with lengths $TW_{out}$, $T\overset{L}{\underset{in}{W}}$, and $T\overset{S}{\underset{in}{W}}$, respectively.

The outflow sequence sample b(b=0,1,2,⋯) is defined as:


$$\overset{out}{\underset{b}{X}}=\left\{\overset{out}{\underset{i}{x}},\overset{out}{\underset{i+1}{x}},\cdots ,\overset{out}{\underset{i+TW_{out}-1}{x}}\right\}\in {\mathbb{R}}^{N\times TW_{out}}.$$ (4)

The short-term inflow sequence sample b is defined as:


$$\overset{S_{in}}{\underset{b}{X}}=\left\{\overset{in}{\underset{v}{x}},\overset{in}{\underset{v+1}{x}},\cdots ,\overset{in}{\underset{v+T\overset{S}{\underset{in}{W}}-1}{x}}\right\}\in {\mathbb{R}}^{N\times T\overset{S}{\underset{in}{W}}}.$$ (5)

The long-term inflow sequence sample b is defined as:


$$\overset{L_{in}}{\underset{b}{X}}=\left\{\overset{in}{\underset{j}{x}},\overset{in}{\underset{j+1}{x}},\cdots ,\overset{in}{\underset{j+T\overset{L}{\underset{in}{W}}-1}{x}}\right\}\in {\mathbb{R}}^{N\times T\overset{L}{\underset{in}{W}}}.$$ (6)

Where $T\overset{S}{\underset{in}{W}}<T\overset{L}{\underset{in}{W}}$ and v>j. During sample construction, the alignment constraint $v+T\overset{S}{\underset{in}{W}}-1=j+T\overset{L}{\underset{in}{W}}-1=i+TW_{out}-1$ is enforced to incorporate the most recent inflow information while avoiding future inflow leakage.

OD data are derived from complete individual travel records and inherently reflect short-term passenger movements between origins and destinations. Accordingly, each OD matrix sample is designed to encode spatiotemporal station-level associations within a short time window, enabling effective exploitation of OD information. Specifically, for each short-term inflow sequence sample b, all OD matrices within the interval $\left[v,\;v+T\overset{S}{\underset{in}{W}}-1\right]$ are temporally aggregated, and their sum is used as the corresponding OD matrix sample b, as defined in Eq. (7). This aggregation provides a unified representation of OD flows over the short-term window, capturing the overall travel intensity between stations during the corresponding period.


$$O_{b}=\left(O_{v}+O_{v+1}+\cdots +O_{v+T\overset{S}{\underset{in}{W}}-1}\right)\in {\mathbb{R}}^{N\times N}.$$ (7)

Notably, the OD matrix sample is not constructed as a temporal sequence. Instead, it is integrated as an input feature alongside the short-term inflow sequence. This design preserves the salient temporal information in the OD data while avoiding the computational overhead of explicit sequential OD representations.

For sample b, the model produces an outflow prediction. The corresponding ground‑truth outflow is given by Eq. (8).


$$\overset{out}{\underset{b}{y}}=\overset{out}{\underset{i+TW_{out}}{x}}\in {\mathbb{R}}^{N}.$$ (8)

Definition 4 (objective equation).

The outflow prediction equation is defined as follows:


$$\overset{pred}{\underset{b}{y}}=f\left(\overset{out}{\underset{b}{X}},\overset{S_{in}}{\underset{b}{X}},\overset{L_{in}}{\underset{b}{X}},O_{b};\Theta \right).$$ (9)

Where f denote the proposed DTIOD model and Θ its learnable parameters, and $\overset{pred}{\underset{b}{y}}\in {\mathbb{R}}^{N}$ represents the one-step-ahead prediction for sample b.

### 2.2 Model construction

**Fig 1:**
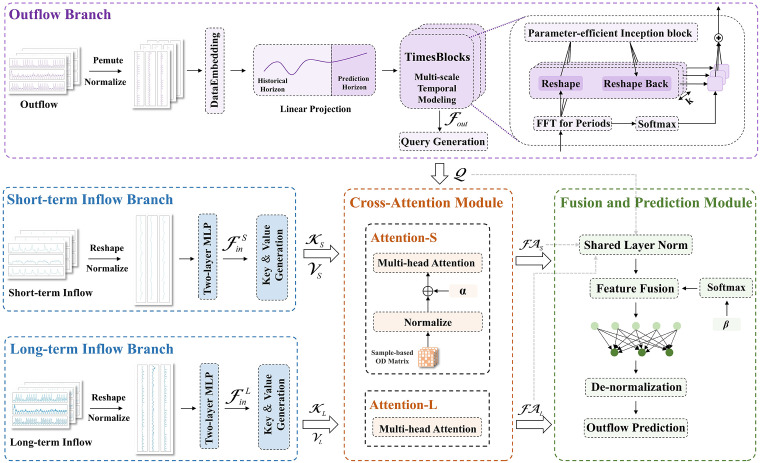
Architecture of the DTIOD model. In the model implementation, let B denote the batch size. The model takes four input tensors: the outflow tensor ${𝒳}^{out}\in {\mathbb{R}}^{B\times N\times TW_{out}}$, the short-term inflow tensor ${𝒳}^{S_{in}}\in {\mathbb{R}}^{B\times N\times T\overset{S}{\underset{in}{W}}}$, the long-term inflow tensor ${𝒳}^{L_{in}}\in {\mathbb{R}}^{B\times N\times T\overset{L}{\underset{in}{W}}}$, and the OD tensor $𝒪\in {\mathbb{R}}^{B\times N\times N}$.

The DTIOD model comprises a feature extraction module, a dual-temporal cross-attention module, and a fusion and prediction module. The feature extraction module contains three parallel branches that encode the outflow, short-term inflow, and long-term inflow sequences into high-dimensional feature representations. The cross-attention module consists of two multi-head attention mechanisms that model the effects of short-term and long-term inflows on the target outflow, thereby capturing multi-scale dependencies without relying on station correlation graphs or explicit spatial modules. The representations from the outflow branch and the cross-attention module are then adaptively fused and passed to a prediction head to generate the final outflow forecasts. The overall architecture is illustrated in Fig 1.

#### 2.2.1 Feature extraction module.

The feature extraction module learns temporal representations of the target outflow and encodes inflow sequences for subsequent dependency modeling. As accurate outflow prediction is the primary objective, the outflow encoder is designed to effectively capture complex temporal dynamics. In contrast, the inflow encoder plays an auxiliary role by supplying Key–Value representations for the cross-attention mechanism. Accordingly, it focuses on producing embeddings that are feature-dimension aligned with the outflow representations, rather than independently modeling detailed inflow dynamics.

Using identical architectures for inflow and outflow feature extraction typically forces a trade-off between representational capacity and computational efficiency. Overly simple designs may fail to capture the complex temporal dynamics of outflow, whereas more expressive ones substantially increase model complexity and computational cost. To address this issue, we adopt an asymmetric feature extraction scheme that tailors modeling capacity to the distinct functional roles of inflow and outflow, thereby preserving predictive performance while maintaining a lightweight architecture.

Specifically, TimesNet [40] is employed to encode outflow temporal features. Based on the TimesBlock architecture, it transforms one-dimensional time series into multi-periodic two-dimensional representations and integrates Inception-style multi-scale convolutional structures to capture temporal variations across different periodic scales. In contrast, inflow sequences are encoded by a lightweight two‑layer multilayer perceptron (MLP) [41], which provides sufficiently expressive Key–Value representations for the subsequent cross‑attention module while incurring minimal computational overhead.

(1) Outflow Branch

The input outflow tensor ${𝒳}^{out}\in {\mathbb{R}}^{B\times N\times TW_{out}}$ is first permuted to ${\overset{―}{𝒳}}^{out}\in {\mathbb{R}}^{B\times TW_{out}\times N}$ and normalized along the temporal dimension using Z-score standardization. The normalized sequence is then projected into a $d_{model}$ -dimensional embedding space, yielding the embedded representation $ℰ\in {\mathbb{R}}^{B\times TW_{out}\times d_{model}}$. A linear projection layer subsequently expands the temporal dimension from $TW_{out}$ to $TW_{out}+P$, where P=1 in this study. The resulting tensor is fed into a stack of TimesBlocks to capture complex temporal variations.

Within each TimesBlock, the input is transformed along the temporal dimension using a Fast Fourier Transform (FFT) to obtain its amplitude spectrum. The spectrum is averaged over the batch and embedding dimensions, and the k frequency components with the highest energy are selected and mapped to their corresponding dominant time periods. For each identified period, the temporal axis is reorganized according to the period length, producing a two-dimensional representation. The resulting periodic representations are processed by an Inception-style multi-scale convolution module, which extracts fine-grained patterns within individual periods as well as broader variations across periods. The convolution outputs associated with different periods are weighted and fused using softmax-normalized spectral energies as period-specific importance coefficients. The fused representation is then passed through a linear projection and integrated into the residual connection, a design that strengthens feature preservation and promotes stable gradient propagation. Before being forwarded to the next TimesBlock, Layer Normalization is applied to ensure consistent feature distributions across layers.

The outflow temporal modeling stage outputs a feature sequence ${ℱ}_{out}\in {\mathbb{R}}^{B\times TW_{out}+1\times d_{model}}$. From this sequence, the feature vector at the target prediction step, $\overset{pred}{\underset{out}{ℱ}}\in {\mathbb{R}}^{B\times d_{model}}$, is extracted to construct the Query. This vector is linearly projected to an intermediate representation $ℐ\in {\mathbb{R}}^{B\times Nd_{model}}$ and reshaped into $𝒬\in {\mathbb{R}}^{B\times N\times d_{model}}$, resulting in one Query vector for each station.

(2) Inflow Branch

The inflow branch comprises short-term and long-term components that share an identical architecture but operate at different temporal scales. Given an input tensor ${𝒳}^{in}\in {\mathbb{R}}^{B\times N\times TW_{in}}$, where $TW_{in}\in \left\{T\overset{S}{\underset{in}{W}},T\overset{L}{\underset{in}{W}}\right\}$, each sample is first normalized along the temporal dimension using Z-score standardization. The normalized tensor is reshaped into ${\tilde{𝒳}}^{in}\in {\mathbb{R}}^{BN\times TW_{in}}$, treating the inflow series of each station as an independent temporal sequence. These sequences are projected into a $d_{model}$ -dimensional feature space through a two-layer MLP, producing embeddings that are channel‑aligned with the corresponding outflow features. The mapping is defined as follows:


$${\text{MLP}}_{\text{S}}:T\overset{S}{\underset{in}{W}}\to \overset{S}{\underset{hidden}{d}}\to d_{model},$$ (10)

$${\text{MLP}}_{\text{L}}:T\overset{L}{\underset{in}{W}}\to \overset{L}{\underset{hidden}{d}}\to d_{model}.$$ (11)

The mapped features are reshaped to obtain $\overset{S}{\underset{in}{ℱ}}\in {\mathbb{R}}^{B\times N\times d_{model}}$ and $\overset{L}{\underset{in}{ℱ}}\in {\mathbb{R}}^{B\times N\times d_{model}}$. These tensors are then transformed by separate linear layers followed by layer normalization to produce the corresponding Key and Value representations, as defined in Eqs. (12)–(13).


$${𝒦}_{S}=\text{LayerNorm}\left(\overset{\text{S}}{\underset{\text{proj}}{\text{K}}}\left(\overset{S}{\underset{in}{ℱ}}\right)\right),\;{𝒱}_{S}=\text{LayerNorm}\left(\overset{\text{S}}{\underset{\text{proj}}{\text{V}}}\left(\overset{S}{\underset{in}{ℱ}}\right)\right),$$ (12)

$${𝒦}_{L}=\text{LayerNorm}\left(\overset{\text{L}}{\underset{\text{proj}}{\text{K}}}\left(\overset{L}{\underset{in}{ℱ}}\right)\right),\;{𝒱}_{L}=\text{LayerNorm}\left(\overset{\text{L}}{\underset{\text{proj}}{\text{V}}}\left(\overset{L}{\underset{in}{ℱ}}\right)\right).$$ (13)

#### 2.2.2 Dual-temporal cross-attention module.

**Fig 2:**
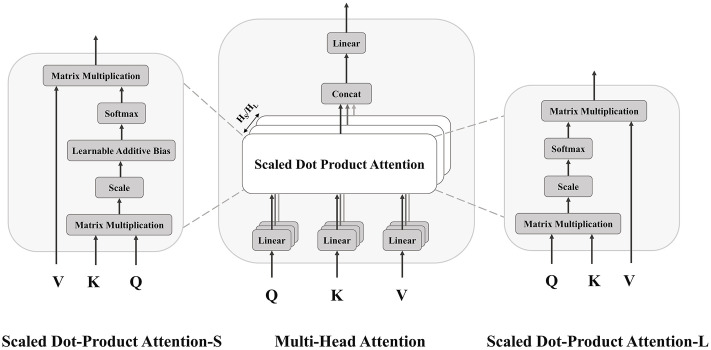
Dual-temporal cross-attention module for inflow–outflow dependency modeling.

The cross-attention module comprises two independent multi-head attention mechanisms, denoted as Attention-L and Attention-S, as shown in Fig 2. Each mechanism uses the outflow features as the Query. Attention-L uses long-term inflow features as the Key and Value, enabling the outflow Query to aggregate long-term contextual information and encode semantic dependencies relevant to the target outflow. In contrast, Attention-S uses the short-term inflow features as Key and Value, incorporating a learnable scaling OD tensor $𝒪\in {\mathbb{R}}^{B\times N\times N}$ as an additive bias to the attention logits. This approach effectively integrates short-term flow dynamics and prior spatiotemporal relationships into the attention mechanism.

(1) Attention-L

Let $H_{\text{L}}$ denote the number of attention heads in Attention-L, such that the dimension of each head is $\overset{L}{\underset{k}{d}}=d_{model}/H_{L}$. For head i, the corresponding projection matrices are denoted as $\overset{Q_{L}}{\underset{i}{W}}\in {\mathbb{R}}^{d_{model}\times \overset{L}{\underset{k}{d}}},\;\overset{K_{L}}{\underset{i}{W}}\in {\mathbb{R}}^{d_{model}\times \overset{L}{\underset{k}{d}}}\text{and}\overset{V_{L}}{\underset{i}{W}}\in {\mathbb{R}}^{d_{model}\times \overset{L}{\underset{k}{d}}}$. The mapping for head i is computed as follows:


$$\overset{L}{\underset{i}{𝒬}}=𝒬\overset{Q_{L}}{\underset{i}{W}},\;\overset{L}{\underset{i}{𝒦}}={𝒦}_{L}\overset{K_{L}}{\underset{i}{W}},\;\overset{L}{\underset{i}{𝒱}}={𝒱}_{L}\overset{V_{L}}{\underset{i}{W}}.$$ (14)

The output of the attention head i is given by:


$$\overset{L}{\underset{i}{\text{head}}}=\text{softmax}\left(\frac{\overset{L}{\underset{i}{𝒬}}{\left(\overset{L}{\underset{i}{𝒦}}\right)}^{\text{T}}}{\sqrt{\overset{L}{\underset{k}{d}}}}\right)\overset{L}{\underset{i}{𝒱}}.$$ (15)

Where $\overset{L}{\underset{i}{𝒬}}{\left(\overset{L}{\underset{i}{𝒦}}\right)}^{\text{T}}$ represents the dot-product attention logits for the head i, $\frac{\text{1}}{\sqrt{\overset{L}{\underset{k}{d}}}}$ is the scaling factor, and softmax(·) is the activation function.

The outputs of all attention heads are concatenated and projected to produce the final output $ℱ{𝒜}_{\;L}$ of Attention-L.


$$ℱ{𝒜}_{\;L}={\text{Attention}}_{\;L}\left(𝒬,{𝒦}_{\;L},{𝒱}_{\;L}\right)=\text{Concat}\left(\overset{L}{\underset{\text{1}}{\text{head}}},\ldots ,\overset{L}{\underset{H_{L}}{\text{head}}}\right)W^{O_{L}}.$$ (16)

Where $W^{O_{L}}\in {\mathbb{R}}^{H_{L}\overset{L}{\underset{k}{d}}\times d_{model}}$.

(2) Attention-S

Let $H_{\text{S}}$ denote the number of attention heads in Attention-S, such that the dimension of each head is $\overset{S}{\underset{k}{d}}=d_{model}/H_{S}$. For head i, the corresponding projection matrices are denoted as $\overset{Q_{S}}{\underset{i}{W}}\in {\mathbb{R}}^{d_{model}\times \overset{S}{\underset{k}{d}}},\;\overset{K_{S}}{\underset{i}{W}}\in {\mathbb{R}}^{d_{model}\times \overset{S}{\underset{k}{d}}}\text{ and }\overset{V_{S}}{\underset{i}{W}}\in {\mathbb{R}}^{d_{model}\times \overset{S}{\underset{k}{d}}}$. The mapping for head i is computed as follows:


$$\overset{S}{\underset{i}{𝒬}}=𝒬\overset{Q_{S}}{\underset{i}{W}},\;\overset{S}{\underset{i}{𝒦}}={𝒦}_{S}\overset{K_{S}}{\underset{i}{W}},\;\overset{S}{\underset{i}{𝒱}}={𝒱}_{S}\overset{V_{S}}{\underset{i}{W}}.$$ (17)

In contrast to Attention-L, Attention-S introduces the OD tensor $𝒪\in {\mathbb{R}}^{B\times N\times N}$ as an additive bias in the attention logits, scaled by a learnable scalar α. To stabilize the attention computation, the OD tensor is normalized using Eqs. (18)–(19).

Each OD matrix $O_{b}\in {\mathbb{R}}^{N\times N}$ within the tensor $𝒪\in {\mathbb{R}}^{B\times N\times N}$ undergoes an element-wise log1p transformation:


$$\overset{pq}{\underset{b}{\varpi }}=log\left(1+\overset{pq}{\underset{b}{O}}\right).$$ (18)

Where $\overset{pq}{\underset{b}{O}}$ represents the passenger flow from station p to station q in sample b.

For each sample b, the resulting matrix ${\tilde{O}}_{b}\in {\mathbb{R}}^{N\times N}$ is then normalized using min-max scaling based on its sample-specific maximum and minimum values, ensuring that all entries fall within the interval [0, 1].


$$\overset{pq}{\underset{b}{\tilde{O}}}=\frac{\overset{pq}{\underset{b}{\varpi }}-m_{b}}{M_{b}-m_{b}}.$$ (19)

Where $m_{b}=\underset{p,q}{min}\left(\overset{pq}{\underset{b}{\varpi }}\right),\;M_{b}=\underset{p,q}{max}\left(\overset{pq}{\underset{b}{\varpi }}\right).$ The normalized matrices are then stacked to form the processed OD tensor ${\tilde{𝒪}}_{b}\in {\mathbb{R}}^{B\times N\times N}$.

The attention logits for head i are expressed as:


$$\overset{S}{\underset{i}{𝒵}}=\frac{\overset{S}{\underset{i}{𝒬}}{\left(\overset{S}{\underset{i}{𝒦}}\right)}^{\text{T}}}{\sqrt{\overset{S}{\underset{k}{d}}}}+exp\left(\alpha \right){\tilde{𝒪}}_{b}.$$ (20)

Here, α>0 is a learnable scalar that adaptively modulates the contribution of the OD bias to the attention logits. It balances the bias against the Query-Key similarity, ensuring that OD information enhances the attention distribution without dominating it. The exp(⋅) function guarantees the scaling factor remains positive, thereby preserving the directionality of the bias.

The output of the attention head i is given by:


$$\overset{S}{\underset{i}{\text{head}}}=\text{softmax}\left(\overset{S}{\underset{i}{𝒵}}\right)\overset{S}{\underset{i}{𝒱}}$$ (21)

The outputs of all attention heads are concatenated and projected to produce the final output $ℱ{𝒜}_{\;S}$ of Attention-S.


$$ℱ{𝒜}_{\;S}={\text{Attention}}_{S}\left(𝒬,{𝒦}_{S},{𝒱}_{S}\right)=\text{Concat}\left(\overset{S}{\underset{\text{1}}{\text{head}}},\ldots ,\overset{S}{\underset{H_{S}}{\text{head}}}\right)W^{O_{S}}$$ (22)

Where $W^{O_{S}}\in {\mathbb{R}}^{H_{S}\overset{S}{\underset{k}{d}}\times d_{model}}$.

#### 2.2.3 Fusion and prediction module.

The feature fusion module adaptively integrates three feature streams, namely 𝒬, $ℱ{𝒜}_{\;S}$, and $ℱ{𝒜}_{\;L}$, using learnable weighting coefficients. Specifically, 𝒬 is the station-level query vector extracted from the outflow temporal feature sequence, representing the contextualized outflow state at the target prediction time step. $ℱ{𝒜}_{\;S}$ and $ℱ{𝒜}_{\;L}$ correspond to the enhanced representations obtained by attending to short-term and long-term inflow features, respectively, thereby capturing their multi-scale influences on the target outflow.

Given the heterogeneous numerical distributions and scales of these feature streams, a shared layer normalization is first applied, producing the normalized representations $\tilde{𝒬}$, $\tilde{ℱ}{𝒜}_{\;L}$, and $\tilde{ℱ}{𝒜}_{\;S}$. A learnable parameter vector $\beta =\left[\beta_{Q},\;\beta_{L},\;\beta_{S}\right]$ is then passed through a Softmax function to generate adaptive fusion weights:


$$w_{i}=\frac{e^{\beta_{i}}}{\sum_{j}e^{\beta_{j}}},\;i,j\in \left\{Q,L,S\right\}.$$ (23)

The final fused representation is computed as the weighted sum of the normalized features:


$${ℱ}_{fused}=w_{Q}\tilde{𝒬}+w_{L}\tilde{ℱ}{𝒜}_{\;L}+w_{S}\tilde{ℱ}{𝒜}_{\;S}.$$ (24)

This design enables the model to adaptively regulate the contribution of passenger flow features from different sources to the final prediction.

The fused representation ${ℱ}_{fused}$ is fed into a linear prediction head $f_{\theta }\left(⬝\right)$ to generate the normalized passenger flow estimate $\overset{pred}{\underset{norm}{𝒴}}\in {\mathbb{R}}^{B\times N}$. The estimate is subsequently re-denormalized using the mean and standard deviation recorded during the outflow-branch normalization stage, restoring it to the original scale and yielding the final station-level passenger flow prediction ${𝒴}^{pred}\in {\mathbb{R}}^{B\times N}$.

## 3 Experimental data and model configuration

### 3.1 Description and partition of the dataset

The performance of DTIOD is evaluated using real-world AFC data collected from the Hangzhou metro system between January 1 and January 25, 2019. The dataset covers four metro lines (Lines 1, 2, 4, and 9) and includes 81 stations, indexed from 0 to 80. Owing to missing entry and exit records, the passenger flow at Station 54 appears as zero in the dataset.

The raw AFC data are systematically cleaned to mitigate random errors arising during data acquisition. The cleaning procedures include: (i) removing records outside operating hours from 5:30–23:30; (ii) discarding incomplete trips that contain only an entry or only an exit record; (iii) eliminating logically inconsistent records, such as those in which the entry time is later than the exit time; and (iv) excluding trips with durations exceeding four hours. This criterion follows the Hangzhou Metro fare policy, which defines a valid trip as one completed within four hours. After data cleaning, a total of 28,868,674 valid and complete trip records remain.

Valid trip records are aggregated to a time granularity of 15 minutes to construct station-level inflow and outflow time series. With a daily operating duration of 18 hours, the system produces 72 time steps per day. Over the 25-day study period, this yields T=1800 consecutive time steps indexed from 0 to 1799. The resulting data form matrices of size N×T, where N=81 denotes the number of stations. The dataset is split chronologically into training, validation, and test sets using ratios of 0.72, 0.08, and 0.20, respectively. Accordingly, the index ranges are 0–1295 for training, 1296–1439 for validation, and 1440–1799 for testing, corresponding to the periods from 1–18 January, 19–20 January, and 21–25 January 2019.

### 3.2 Model configuration and evaluation metrics

The model requires four input tensors, for which the temporal window lengths $TW_{out}$, $T\overset{S}{\underset{in}{W}}$, and $T\overset{L}{\underset{in}{W}}$ must be specified. Here, $TW_{out}$ is set to 72, corresponding to one full day of metro operation, to capture the daily periodicity and time‑varying patterns of outflow while avoiding an excessively long window that would reduce the effective training sample size.

The values of $T\overset{S}{\underset{in}{W}}$ and $T\overset{L}{\underset{in}{W}}$ are determined according to the temporal influence of historical inflow on future outflow. Analysis of the training data indicates that 96.83% of inflow-to-outflow travel times fall within 60 minutes, suggesting that short-term effects are largely confined to this range. At a 15‑minute granularity, this 60‑minute window corresponds to four time steps, and thus $T\overset{S}{\underset{in}{W}}=4$. In contrast, the long-term influence of inflow on outflow exhibits more complex temporal patterns that are not well captured by descriptive statistics. The value of $T\overset{L}{\underset{in}{W}}$ is therefore selected through empirical analysis.

Assume that the maximum temporal span over which historical inflow at station n can influence future outflow at station m is ω. In other words, inflow observed at time step t can affect outflow at most up to time step t+ω. Accordingly, the outflow at time t+ω is influenced by inflow occurring within the interval [t,t+ω], where n and m may be identical or distinct.

Let $\overset{in}{\underset{n}{x}}=\left(\overset{in}{\underset{n,0}{x}},\cdots ,\overset{in}{\underset{n,t}{x}},\cdots ,\overset{in}{\underset{n,T_{train}-1}{x}}\right)\in {\mathbb{R}}^{1\times T_{train}}$ and $\overset{out}{\underset{m}{x}}=\left(\overset{out}{\underset{m,0}{x}},\cdots ,\overset{out}{\underset{m,t}{x}},\cdots ,\overset{out}{\underset{m,T_{train}-1}{x}}\right)\in {\mathbb{R}}^{1\times T_{train}}$ denote the inflow and outflow time series of stations n and m, respectively. Removing the first ω time steps from $\overset{out}{\underset{m}{x}}$ results in the truncated sequence $\overset{out}{\underset{m}{\tilde{x}}}=\left(\overset{\text{out}}{\underset{m,\omega }{x}},\cdots ,\overset{out}{\underset{m,t}{x}},\cdots ,\overset{out}{\underset{m,T_{train}-1}{x}}\right)\in {\mathbb{R}}^{1\times T_{train}-\omega }$. Likewise, removing the last ω time steps from $\overset{in}{\underset{n}{x}}$ leads to the corresponding truncated inflow sequence $\overset{in}{\underset{n}{\tilde{x}}}=\left(\overset{in}{\underset{n,0}{x}},\cdots ,\overset{in}{\underset{n,t}{x}},\cdots ,\overset{in}{\underset{n,T_{train}-1-\omega }{x}}\right)\in {\mathbb{R}}^{1\times T_{train}-\omega }$ produces a set of paired inflow and outflow sequences with different temporal spans, enabling a systematic analysis of the temporal range over which inflow influences outflow. To avoid information leakage and ensure an unbiased evaluation, this paired-sequence construction is performed exclusively on the training set, with no access to validation or test data.

To investigate potential long-term relationships between inflow and outflow, ω is set to cover an entire week of metro operations, taking values from 0 to 503. The Pearson correlation between $\overset{in}{\underset{n}{\tilde{x}}}$ and $\overset{out}{\underset{m}{\tilde{x}}}$ is computed to quantify the variation in their statistical dependence with respect to ω, as defined in Eq. (25).


$$\overset{n,m}{\underset{\omega }{P}}=\frac{\text{Cov}\left(\overset{in}{\underset{n}{\tilde{x}}},\overset{out}{\underset{m}{\tilde{x}}}\right)}{\sigma_{\overset{in}{\underset{n}{\tilde{x}}}}\sigma_{\overset{out}{\underset{m}{\tilde{x}}}}}=\frac{\sum_{t=0}^{T_{train}-1-\omega }\left(\overset{in}{\underset{n,t}{x}}-\overset{―}{\overset{in}{\underset{n}{\tilde{x}}}}\right)\sum_{t=\omega }^{T_{train}-1}\left(\overset{out}{\underset{m,t}{x}}-\overset{―}{\overset{out}{\underset{m}{\tilde{x}}}}\right)}{\sqrt{\sum_{t=0}^{T_{train}-1-\omega }{\left(\overset{in}{\underset{n,t}{x}}-\overset{―}{\overset{in}{\underset{n}{\tilde{x}}}}\right)}^{\text{2}}}\sqrt{\sum_{t=\omega }^{T_{train}-1}{\left(\overset{out}{\underset{m,t}{x}}-\overset{―}{\overset{out}{\underset{m}{\tilde{x}}}}\right)}^{\text{2}}}}.$$ (25)

Where $\overset{―}{\overset{in}{\underset{n}{\tilde{x}}}}$ and $\overset{―}{\overset{out}{\underset{m}{\tilde{x}}}}$ represent the mean values of the sequences $\overset{\text{in}}{\underset{n}{\tilde{x}}}$ and $\overset{\text{out}}{\underset{m}{\tilde{x}}}$, respectively. $\overset{n,m}{\underset{\omega }{P}}$ reflects the correlation between historical inflow at station n and future outflow at station m.

**Fig 3:**
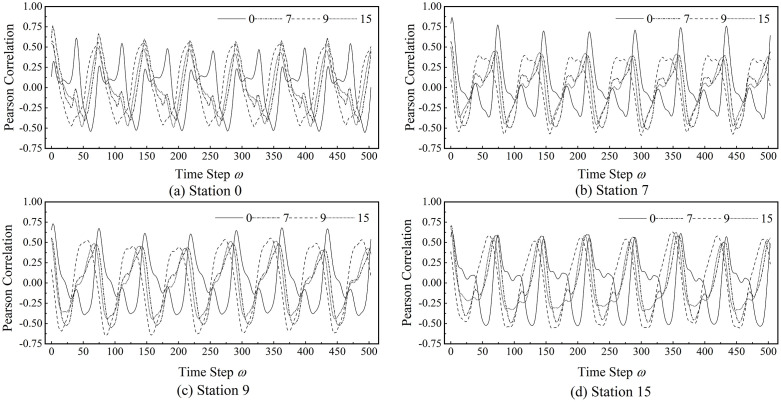
Pearson correlation between inflow and outflow sequences at four stations.

Several stations are randomly selected to examine the association patterns between inflow and outflow sequences using the Pearson correlation coefficient defined above. Fig 3 illustrates the results for stations 0, 7, 9, and 15. Each subplot depicts four curves, showing how the Pearson correlation between the outflow of the target station and the inflow of four stations varies with ω. Although the correlation profiles differ across stations, all exhibit a pronounced periodic structure with a dominant period of approximately 72 time steps. This observation suggests that the long-term influence of inflow on outflow is strongly characterized by daily periodicity. Accordingly, $T\overset{L}{\underset{in}{W}}$ is set to 72.

The configuration of model components and training hyperparameters is summarized below. In the outflow branch, the embedding dimension of the DataEmbedding layer is set to 128, with a dropout rate of 0.2. The temporal encoder comprises two stacked TimesBlocks. Each Inception-style convolution module employs three convolution kernels, with the channel dimension set to 64. The number of key periods is fixed at 2. The two inflow branches share the same architecture. Their feature extraction modules consist of a two-layer MLP, where the first layer uses the ReLU activation function and the second layer adopts GELU. The hidden dimension is set to 128, and a dropout rate of 0.2 is applied between the two layers to improve generalization.

The long-term cross-attention module (Attention-L) employs 8 attention heads with a per-head dimension of 16, while the short-term module (Attention-S) uses 4 heads with a per-head dimension of 32. The learnable scalar α, which controls the scaling of the OD bias, is initialized to 1.11. The parameter vector β, which obtains adaptive fusion weights for the three feature streams via softmax, is initialized as [0.0, 1.0, 1.2].

The model is trained using the AdamW optimizer with a weight decay of 1e-05. The mean squared error (MSE) loss, defined in Eq. (26), is used for training. Gradient clipping with a threshold of 1.0 is applied to prevent exploding gradients. Different learning rates are assigned to different modules. The outflow branch uses an initial learning rate of 0.001. The inflow branches, the cross-attention module, and the learnable parameters α and β adopt a learning rate scaled by a factor γ, which is set to 0.9 relative to the base learning rate. This slight reduction helps stabilize training and allows the primary outflow branch to guide the optimization process. A ReduceLROnPlateau scheduler is applied during training. If the validation root mean squared error (RMSE), defined in Eq. (27), does not improve for three consecutive epochs, the learning rate is reduced by half, with a minimum value of 1e-06. The batch size is set to B=32, and early stopping with a patience of 10 epochs is employed to mitigate overfitting.


$$Loss=MSE=\frac{\text{1}}{B}\sum_{b=0}^{B-1}{\left(\overset{out}{\underset{b}{y}}-\overset{pred}{\underset{b}{y}}\right)}^{\text{2}}$$ (26)

Predictive performance is evaluated using RMSE, mean absolute error (MAE), and weighted mean absolute percentage error (WMAPE), which are defined in Equations (27)–(29).


$$RMSE=\sqrt{MSE}=\sqrt{\frac{\text{1}}{B}\sum_{b=0}^{B-1}{\left(\overset{out}{\underset{b}{y}}-\overset{pred}{\underset{b}{y}}\right)}^{\text{2}}},$$ 27)

$$MAE=\frac{\text{1}}{B}\sum_{b=0}^{B-1}\;\left|\left(\overset{out}{\underset{b}{y}}-\overset{pred}{\underset{b}{y}}\right)\right|,$$ 28)

$$WMAPE=\sum_{b=0}^{B-1}\left(\frac{\overset{out}{\underset{b}{y}}}{\sum_{b=0}^{B-1}\overset{out}{\underset{b}{y}}}\left|\frac{\overset{out}{\underset{b}{y}}-\overset{pred}{\underset{b}{y}}}{\overset{out}{\underset{b}{y}}}\right|\right),$$ 29)

### 3.3 Baseline models

To objectively evaluate the predictive performance of DTIOD, we establish a multilevel comparison framework comprising baseline models from four methodological categories: statistical methods, traditional machine learning models, deep temporal models, and spatiotemporal fusion approaches. These baselines are selected based on their structural relevance to DTIOD and their compatibility with the Hangzhou metro dataset. Detailed model configurations are provided below.

(1) Statistical and traditional machine learning models.

HA (historical average) uses the historical mean passenger flow of each station as the prediction and requires neither model training nor hyperparameter tuning, serving as a basic statistical baseline. RF (random forest) [42] and SVR (support vector regression) [43] are selected as representative machine learning baselines, which model nonlinear input–output relationships through ensemble learning and kernel-based methods, respectively. Both methods remain stable when the sample size is small or the feature dimension is limited. In the experiments, each model uses an input sequence length of 4. RF is implemented with 100 trees. SVR employs a radial basis function kernel, with ε=0.001 and C=5. Comparing DTIOD with HA, RF, and SVR quantifies its performance gains over conventional non‑deep learning methods.

(2) Deep learning temporal models.

Metro passenger flow typically exhibits pronounced peak periods together with stable daily temporal patterns. Accurately capturing short-term fluctuations and long-term periodicity is therefore essential for reliable prediction. LSTM (long short-term memory) [44] and GRU (gated recurrent unit) [45] are adopted as representative deep learning temporal baselines because of their ability to model multi-scale temporal dependencies through gating mechanisms and their extensive use in transportation forecasting studies. In the experiments, both models take a historical sequence of length 36 as input and use a hidden dimension of 128. A fully connected layer with 256 units and a dropout rate of 0.2 is applied before the output layer. TimesNet is a recent temporal model that has demonstrated strong predictive performance and also serves as the temporal encoder in the outflow branch of DTIOD. Using TimesNet as a baseline enables a direct assessment of the performance gains brought by the proposed dual-temporal inflow–outflow dependency modeling. For a fair comparison, the hyperparameters of TimesNet are kept identical to those used in the outflow branch of DTIOD. Comparisons with LSTM, GRU, and TimesNet therefore provide insight into the advantages of DTIOD over purely temporal modeling approaches.

(3) Spatiotemporal fusion models.

Metro networks inherently exhibit graph structure. As a result, spatiotemporal fusion models based on graph convolution have been widely applied in passenger flow prediction studies. However, the Hangzhou Metro dataset used in this study contains missing passenger flow records at a station. Such data incompleteness may disrupt feature propagation in models that rely on predefined graph structures. To examine the impact of this issue, several representative graph-based models are selected as comparison baselines, including STGCN [46] (spatio-temporal graph convolutional networks), PVGCN [15] (physical-virtual collaboration graph network), PMR-GCN [29] (regularized spatial-temporal graph convolutional networks for metro passenger flow prediction), and MR-STN [33] (spatio-temporal network framework based on multi-relational). These models enable an evaluation of graph structured modeling approaches under the data conditions of the Hangzhou Metro dataset. Among these models, STGCN is a classical fusion framework that jointly models spatial and temporal dependencies through graph and temporal convolutions. PVGCN integrates physical and virtual network topologies to characterize passenger flow dynamics from multiple graph perspectives. PMR-GCN combines personalized graph convolution with multi-head self-attention to model complex spatiotemporal dependencies. MR-STN explicitly models the coupling between inflow and outflow and extracts local and global features through an enhanced STFL (spatio-temporal feature learner) module.

Comparisons with STGCN, MR-STN, PVGCN, and PMR-GCN enable a systematic evaluation of the predictive performance of DTIOD as a graph-free model. In particular, comparisons with MR-STN and PVGCN provide insight into different strategies for utilizing OD information. MR-STN employs time-step-level OD data to characterize the relationship between inflow and outflow, whereas PVGCN relies on globally aggregated static OD data to construct a station correlation graph. Considering these approaches together helps clarify how different forms of OD information influence model performance and facilitates an assessment of the relative effectiveness of DTIOD within this research line.

In addition, the graph-free model SDT-GRU [35] is included as an additional baseline. SDT-GRU adopts a sequence-to-sequence architecture built upon stacked DT-GRU layers, where each DT-GRU integrates a dual-branch Transformer module into the GRU to capture spatiotemporal dependencies without relying on predefined graphs. Comparing DTIOD with SDT-GRU, which follows a similar graph-free modeling paradigm, helps reveal performance differences within this framework. All spatiotemporal fusion models considered in this study adopt the parameter settings recommended in their original publications.

All models are configured for single-step passenger flow prediction. Deep learning models are trained with an early stopping strategy (patience = 10) to mitigate overfitting. All implementations are based on the PyTorch framework. Each model is evaluated over five independent runs, and the reported results are averaged across runs.

## 4 Case analysis

This section evaluates the DTIOD model along four dimensions: overall performance, training efficiency, prediction accuracy, and ablation analysis.

### 4.1 Overall performance analysis

Table 1 summarizes the overall performance of DTIOD and all baselines on the full test set. For each deep learning model, it also reports the average training time per epoch and the total training time.

**Table 1:** Prediction accuracy and training time of compared models.

Models	Metrics			Average epoch time/ (s)	Total training time/ (s)
	RMSE	MAE	WMAPE/ %		
HA	186.7030	107.9209	49.1995	–	–
RF	43.2451	24.1771	11.2841	–	–
SVR	42.0693	25.6468	11.9424	–	–
LSTM	44.2563	25.0160	11.7817	0.7768	52.8200
GRU	41.9027	24.7607	11.6222	0.8135	70.7783
STGCN	44.8551	25.3269	11.8749	1.2844	71.9283
MR-STN	51.3041	28.0072	18.9286	5.6089	1261.9984
PVGCN	43.7384	25.8464	11.3894	68.9589	2482.5206
SDT-GRU	39.4306	24.1805	10.6553	6.3675	503.0350
PMR-GCN	67.2293	38.5539	17.9772	8.6128	241.1591
TimesNet	41.2489	24.6916	11.4398	1.1387	50.1019
DTIOD	35.1919	21.3750	9.9268	1.1646	66.3849

In general, predictive accuracy is expected to follow a hierarchical pattern, with spatiotemporal fusion models outperforming purely temporal models, deep learning approaches surpassing traditional machine learning methods, and the latter exceeding statistical baselines. The results in Table 1, however, deviate from this pattern. While the simple statistical baseline (HA) performs worst, traditional machine learning models (RF and SVR) achieve higher accuracy than several deep learning approaches. In addition, purely temporal models, including LSTM, GRU, and TimesNet, outperform most spatiotemporal fusion models across the evaluated metrics.

A joint analysis of model architectures and experimental results reveals a consistent trend that greater reliance on graph-based spatial modeling corresponds to degraded predictive performance. Although STGCN, MR-STN, PVGCN, and PMR-GCN achieve strong results on several public metro datasets, their performance deteriorates markedly on the Hangzhou Metro dataset. This decline stems from the complete absence of entry and exit records at Station 54, which disrupts adjacency-based feature aggregation in graph convolutional layers. Because graph convolution relies on coherent feature propagation across connected nodes, missing node features hinder spatial information diffusion. This structural deficiency introduces noise into the learned representations, weakens spatial feature extraction, and ultimately reduces forecasting accuracy. These results suggest that spatiotemporal fusion models heavily dependent on predefined graph structures exhibit limited robustness when node features are incomplete or data quality is inconsistent.

Among all baseline methods, SDT-GRU achieves the highest predictive accuracy. Its strong performance is primarily attributed to its ability to capture global spatial correlations and temporal dependencies without relying on a predefined station correlation graph, thereby alleviating the adverse effects of missing passenger flow records. Following the same graph-free modeling paradigm, DTIOD further outperforms SDT-GRU across all evaluation metrics, achieving average reductions of 10.75%, 11.60%, and 6.84% in RMSE, MAE, and WMAPE, respectively. Beyond predictive accuracy, DTIOD also exhibits clear advantages in training time. As indicated by the last two columns of Table 1, its average iteration time per epoch is only marginally higher than that of purely temporal models, while its total training time is substantially lower than that of other spatiotemporal fusion models and even shorter than that of the GRU baseline. Although DTIOD performs well in terms of accuracy and training time, a more detailed examination of its training efficiency, complexity and convergence behavior remains worthwhile.

### 4.2 Comparison of model complexity and training efficiency

**Fig 4:**
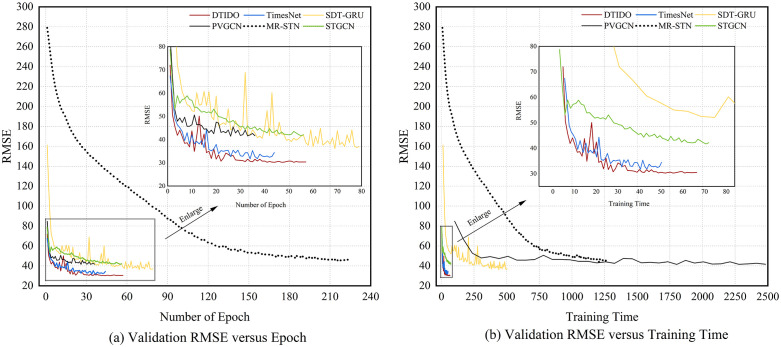
Training dynamics of DTIOD and baselines.

Considering the structural characteristics and predictive performance of the baseline models, five representative approaches are selected for comparison in terms of model complexity and training efficiency: TimesNet, SDT-GRU, STGCN, MR-STN, and PVGCN. Model complexity is evaluated using the number of parameters and FLOPs (floating-point operations). These models represent different modeling paradigms and information utilization strategies. TimesNet and SDT-GRU belong to graph-free architectures, whereas STGCN, MR-STN, and PVGCN are graph-based models. Fig 4 presents the comparison between DTIOD and the selected baselines, illustrating the evolution of validation errors with respect to training epochs and cumulative training time. The corresponding statistics for model parameters and FLOPs, computed with a batch size of 1, are reported in Table 2.

**Table 2:** Comparison of model parameters and FLOPs.

Model	DTIDO	TimesNet	SDT-GRU	PVGCN	MR-STN	ST-GCN
Parameters (Million)	2.7823	1.1953	1.5500	37.9930	1.1615	0.1801
FLOPs (Million)	199.0802	187.8368	10698.3964	927423.1728	1639.7880	30.9181

Graph-based models generally exhibit lower training efficiency, although substantial differences exist among them. Examination of Tables 1 and 2 together with Fig 4 reveals several patterns. STGCN adopts a relatively simple graph architecture and therefore has the lowest computational complexity among the graph-based models. Its training time is comparable to that of the graph-free models TimesNet and DTIOD. However, the error curve of STGCN gradually flattens during the later stages of training, indicating limited potential for further improvement. Among the graph-based models, MR-STN and PVGCN employ multi-graph modeling structures and consequently have much higher computational complexity than STGCN. In particular, PVGCN requires 68.9589 seconds for a single training epoch, which is approximately 54 times longer than that of STGCN. Its parameter count and FLOPs are also the largest among all compared models. By contrast, MR-STN has moderate computational complexity, but its error decreases relatively slowly during training, resulting in low training efficiency. The model ultimately requires 1261.9984 seconds to complete training and produces the largest prediction error among all comparison methods.

Graph-free models generally demonstrate advantages in training efficiency, although differences remain among them. TimesNet, as a purely temporal model, requires a total training time of 50.1019 seconds. Its computational complexity is only higher than that of STGCN and remains relatively low among all compared models. However, its predictive accuracy is limited and shows signs of overfitting. TimesNet achieves lower validation error than SDT-GRU but performs worse on the test set, indicating weaker generalization ability. By contrast, although SDT-GRU also follows a graph-free modeling paradigm, its computational complexity is second only to that of PVGCN. The model adopts a sequence-to-sequence architecture, which restricts parallel computation. As a result, its training time per epoch and total training time are substantially higher than those of other graph-free models and even exceed those of some graph-based models such as STGCN. These results indicate that the use of graph structures is not the sole factor determining model training efficiency.

As a graph-free architecture, DTIOD achieves a favorable trade-off between computational cost and predictive accuracy. The total training time is 66.3849s, which is only 24.53% higher than that of TimesNet, while the predictive accuracy surpasses all baseline models. The computational complexity remains low and is only slightly higher than that of TimesNet. During training, the error curve shows moderate fluctuations in the intermediate stage, after which the RMSE decreases rapidly and converges to a stable level, indicating efficient optimization. The superior performance of DTIOD stems from the coordinated design of its architecture and information utilization strategy. First, DTIOD inherits the multiscale temporal modeling capability of TimesNet and further introduces inflow–outflow dependency modeling, which significantly enhances predictive accuracy. The substantial performance gap between DTIOD and TimesNet observed in the experiments confirms the importance of explicitly modeling inflow and outflow dependencies. Second, the overall architecture remains simple because the model does not rely on graph structures or spatial modules, which helps maintain low computational complexity. Third, DTIOD incorporates OD information through a sample-level OD matrix used as an attention bias, which enables the integration of OD dynamics with limited computational overhead. In contrast, MR-STN models the OD matrix at the time-step level and therefore introduces substantially higher computational complexity. PVGCN instead constructs station correlation graphs based on static OD information, which limits its ability to capture dynamic passenger flow patterns.

### 4.3 Prediction performance analysis

**Fig 5:**
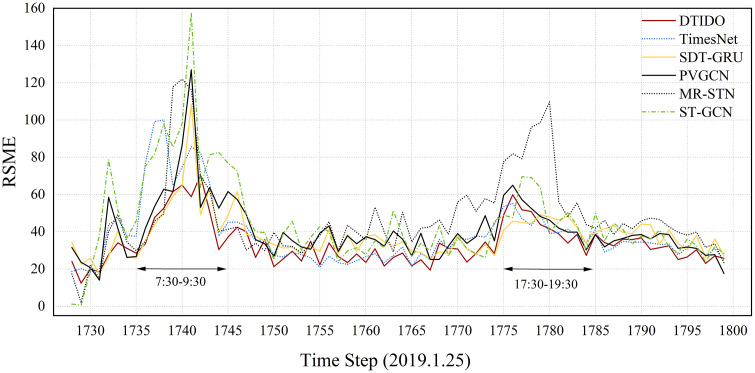
Prediction error comparison between DTIOD and baselines.

This section evaluates DTIOD’s prediction performance across temporal and station-level dimensions, relative to five baseline models. Fig 5 presents the prediction errors at each time step on 25 January 2019. All models exhibit a bimodal error distribution over time. STGCN, MR-STN, and PVGCN show the largest error fluctuations, whereas SDT-GRU and TimesNet demonstrate comparatively greater stability. DTIOD yields the smoothest error trajectory and achieves lower errors than the baseline models across most time steps.

Table 3 further reports the prediction errors of each model during peak and off-peak periods, measured by RMSE. The results show that all models produce larger errors during peak periods than during off-peak periods. This pattern mainly arises because passenger flows during peak hours are both larger in magnitude and more volatile. The resulting increase in prediction error therefore reflects higher demand intensity and greater uncertainty in passenger flows rather than a systematic bias of model parameters toward specific time periods. In terms of individual model performance, DTIOD achieves the lowest prediction error during both the off-peak period and the morning peak period, outperforming all comparison models overall. During the evening peak period, its error is slightly higher than that of TimesNet. Notably, the prediction error of TimesNet during the morning peak period is substantially higher and is lower only than that of STGCN. Considering results across all time periods, DTIOD maintains consistently high prediction accuracy under different temporal scenarios, demonstrating strong robustness and generalization capability.

**Table 3:** Model prediction errors across time periods.

Time Periods	DTIDO	TimesNet	SDT-GRU	PVGCN	MR-STN	ST-GCN
Off-peak	29.0793	31.8676	34.1374	35.6150	39.3890	37.5018
Morning peak	55.0671	81.2395	59.2742	68.7792	77.3198	92.6402
Evening peak	44.9392	44.1308	46.1503	48.8686	78.1508	53.6109

At the station level, DTIOD’s performance is assessed at four representative stations. Station 33 is a terminal station on Line 9, characterized by typical peak-hour commuting patterns. Station 46 serves as an interchange between Lines 2 and 4 and exhibits more complex passenger-flow dynamics. Station 15, the busiest multimodal hub in the network, handles a diverse range of passenger flows. Finally, Station 55, located adjacent to Station 54 where data are missing, provides a test of DTIOD’s robustness in handling local data incompleteness.

**Fig 6:**
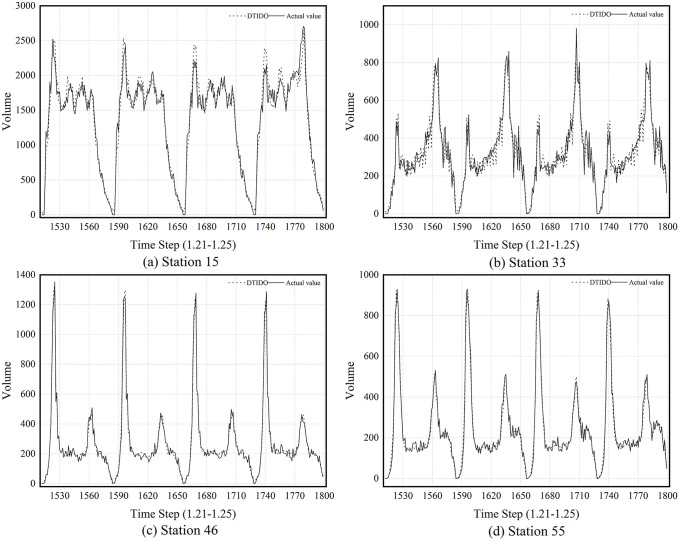
Predicted vs. actual outflow at four representative stations.

Fig 6 compares the predicted and actual outflow at the four representative stations. As shown in Figs 6(c) and 6(d), DTIOD achieves high accuracy at stations such as 46 and 55, where passenger flow patterns are relatively regular, with the predicted curves closely matching the ground-truth trajectories. The results for Station 55 further demonstrate that missing data at a neighboring station do not significantly impair model performance. In contrast, predictions for Stations 15 and 33, shown in Figs 6(a) and 6(b), are less accurate. These stations exhibit frequent and pronounced fluctuations in passenger flow, resulting in more complex temporal patterns that increase the difficulty of modeling rapidly changing behaviors. Nevertheless, DTIOD successfully captures the overall trends and responds effectively to sudden changes, demonstrating its robustness and adaptability in complex operating environments.

### 4.4 Ablation study

As described in Section 2.2, DTIOD comprises two core components. TimesNet is responsible for extracting temporal features from outflow, while a cross-attention mechanism models the multi-scale temporal influence of inflow on outflow. Ablating this cross-attention mechanism reduces DTIOD to the TimesNet baseline; their comparative predictive performance has been thoroughly analyzed in earlier sections and is not revisited here. To systematically evaluate the contribution of the remaining components in DTIOD, we conduct a series of ablation experiments by modifying the model architecture and feature fusion strategy. The corresponding results are summarized in Table 4.

**Table 4:** Prediction performance of DTIOD ablation variants.

		Metrics				Metrics	
Models	RMSE	MAE	WMAPE/ %	Models	RMSE	MAE	WMAPE/ %
DTIOD-Only Attention-L	37.3948	22.7035	10.5509	DTIOD-No Attention-L	36.3183	21.6812	10.0577
DTIOD-Only Attention-S	37.6893	23.0059	10.6003	DTIOD-No Attention-S	37.1705	22.6342	10.4393
DTIOD-Only Attention	37.3625	22.6422	10.4732				

The ablation variants are defined as follows.

(1) DTIOD-Only-Attention-L retains only the long-scale attention output $\tilde{ℱ}{𝒜}_{\;L}$ for prediction, with the short-scale attention output $\tilde{ℱ}{𝒜}_{\;S}$ and the query representation $\tilde{𝒬}$ removed.
(2) DTIOD-Only-Attention-S retains only $\tilde{ℱ}{𝒜}_{\;S}$, excluding $\tilde{ℱ}{𝒜}_{\;L}$ and $\tilde{𝒬}$ from the prediction process.
(3) DTIOD-Only-Attention removes the query representation $\tilde{𝒬}$ and directly fuses $\tilde{ℱ}{𝒜}_{\;S}$ and $\tilde{ℱ}{𝒜}_{\;L}$ for prediction.
(4) DTIOD-No-Attention-L removes the long-scale attention module and fuses the short-scale feature $\tilde{ℱ}{𝒜}_{\;S}$ with the query representation $\tilde{𝒬}$ for prediction.
(5) DTIOD-No-Attention-S removes the short-scale attention module and combines $\tilde{ℱ}{𝒜}_{\;L}$ with $\tilde{𝒬}$ before prediction.

For experiments (3) to (5), the feature fusion strategy is as follows:


$${ℱ}_{fused\_3}=\text{sigmoid}\left(\sigma \right)\cdot \tilde{ℱ}{𝒜}_{\;S}+\left(1-\text{sigmoid}\left(\sigma \right)\right)\cdot \tilde{ℱ}{𝒜}_{\;L},$$ (30)

$${ℱ}_{fused\_4}=\text{sigmoid}\left(\sigma \right)\cdot \tilde{𝒬}+\left(1-\text{sigmoid}\left(\sigma \right)\right)\cdot \tilde{ℱ}{𝒜}_{\;S},$$ (31)

$${ℱ}_{fused\_5}=\text{sigmoid}\left(\sigma \right)\cdot \tilde{𝒬}+\left(1-\text{sigmoid}\left(\sigma \right)\right)\cdot \tilde{ℱ}{𝒜}_{\;L},$$ (32)

Here, σ is a learnable parameter, constrained to lie between 0 and 1 after the sigmoid transformation. Its initial value is set to 0.5.

The ablation results in Table 4 confirm that removing any core module degrades performance, validating the necessity of each component in DTIOD. Variants DTIOD-Only-Attention-L and DTIOD-Only-Attention-S produce comparable errors, which are substantially higher than those of other configurations. This result indicates that a single attention branch cannot sufficiently capture the characteristics of outflow. Although DTIOD-Only-Attention achieves slightly improved accuracy, its performance remains limited, highlighting the critical role of the Query component in providing a temporal representation of the target prediction step. In comparison, DTIOD-No-Attention-L and DTIOD-No-Attention-S outperform the single-branch variants, demonstrates that retaining the query representation while removing only one attention branch is more effective than relying on a single inflow attention branch alone. Notably, DTIOD-No-Attention-S exhibits higher errors than DTIOD-No-Attention-L, suggesting that the short-term inflow attention branch has a greater contribution to accurate forecasting.

Overall, the ablation studies provide systematic evidence for the effectiveness of the Attention-L, Attention-S, and Query components in DTIOD. The results indicate that combining multi-scale temporal dependency modeling between inflow and outflow with explicit temporal representations at the target prediction step is crucial for improving outflow forecasting accuracy. Moreover, a comparison with the results in Table 1 shows that although removing individual components leads to some performance degradation, all DTIOD variants consistently outperform every baseline model evaluated in this study. This consistent advantage demonstrates the structural robustness of the proposed framework.

### 4.5 Parameter sensitivity analysis

To evaluate the sensitivity of DTIOD to hyperparameter choices, twelve key parameters are examined, each with six candidate values. The model dimensions include $d_{model}$ as the embedding dimension, $d_{ff}$ as the convolution channel dimension in the outflow branch, $\overset{L}{\underset{hidden}{d}}$ as the hidden dimension in the long-term inflow branch, and $\overset{S}{\underset{hidden}{d}}$ as the hidden dimension in the short-term inflow branch. These parameters are tested over {16, 32, 64, 128, 256, 512}. Architectural parameters include K as the number of convolution kernels in the outflow Inception block, $L_{TB}$ as the number of TimesBlocks in the outflow branch, and TK as the number of key periods in the outflow branch. These parameters are evaluated over {1, 2, 3, 4, 5, 6}. The attention head numbers $H_{\text{L}}$ and $H_{\text{S}}$ are selected from {1, 2, 4, 8, 16, 32}. The scaling factor γ is tested over {0.5, 0.7, 0.9, 1.0, 1.2, 1.5}, while α is evaluated over {0.89, 0.90, 1.00, 1.10, 1.11, 1.20}. The learnable parameter vector β is initialized with six configurations: [0, 0, 0], [1.2, 1, 0], [1, 1.2, 0], [1.2, 0, 1], [0, 1.2, 1], and [0, 1, 1.2].

The candidate values of α are chosen to lie close to 1, as this parameter governs the initial OD bias scaling strength. If α is excessively large, the OD bias dominates the Attention-S computation and weakens the attention structure derived from query–key similarity. If α is too small, the bias contributes little to attention weight allocation. Therefore, α requires finer calibration than the other parameters.

When evaluating an individual parameter, all others are fixed at the default settings defined in Section 3.2. The experimental results are summarized in Table 5. The second column reports the RMSE on the test set under the six candidate settings for each parameter, while the third column presents the corresponding relative changes of RMSE. The relative change (RC) is used to quantify the overall variation in prediction error and is defined as follows.

**Table 5:** Sensitivity analysis of key parameters in DTIOD.

Parameter	RMSE	RC/ %
$d_{model}$	36.6471, 37.3295, 36.1330, 35.1919, 36.7230, 36.7427	5.8627
$d_{ff}$	36.2971, 36.0236, 35.1919, 35.7782, 36.0076, 37.2764	5.7749
$\overset{L}{\underset{hidden}{d}}$	38.2279, 35.9609, 36.0547, 35.1919, 36.1970, 35.6306	8.3843
$\overset{S}{\underset{hidden}{d}}$	37.2718, 37.7443, 35.7148, 35.1919, 37.0324, 35.2175	7.0194
K	36.3844, 36.0617, 35.1919, 35.7025, 36.1108, 35.5722	3.3275
$L_{TB}$	36.1627, 35.1919, 36.0328, 35.9242, 37.3360, 37.1339	5.9071
TK	36.2570, 35.1919, 36.1193, 38.3094, 36.3523, 36.7372	8.5424
$H_{\text{L}}$	36.0477, 36.0721, 35.7500, 35.1919, 36.3122, 35.2081	3.1325
$H_{\text{S}}$	36.5206, 35.6378, 35.1919, 35.8363, 35.8380, 35.4672	3.7168
γ	35.7767, 35.8999, 35.1919, 35.7149, 36.8646, 35.7949	4.6627
α	36.2779, 35.5710, 35.5674, 36.2335, 35.1919, 35.5090	3.0399
β	35.9189, 35.3937, 35.8117, 35.9094, 35.5488, 35.1919	2.0405


$$\text{RC}=\frac{x_{max}-x_{min}}{\overset{―}{x}}\times 100\%.$$ (33)

Here, $x_{max}$ denotes the maximum RMSE, $x_{min}$ denotes the minimum RMSE, and $\overset{―}{x}$ represents the mean RMSE across all candidate settings.

As shown in Table 5, DTIOD maintains stable predictive performance across the examined parameter ranges. Even under less favorable settings, it consistently outperforms all baseline models, demonstrating strong robustness. Further analysis shows that the model is more sensitive to $d_{model}$, $d_{ff}$, $\overset{L}{\underset{hidden}{d}}$, $\overset{S}{\underset{hidden}{d}}$, $L_{TB}$ and TK, as reflected by the relatively large changes in RMSE. Increasing these parameters enhances the feature representation capacity of the model and reduces prediction error. However, beyond an appropriate range, the resulting increase in model complexity raises the risk of overfitting, which may cause the prediction accuracy to plateau or decline. In contrast, the variations in RMSE associated with γ, α, and β remain consistently small, indicating that the model is less sensitive to these training hyperparameters. Therefore, the predictive performance of DTIOD does not critically depend on their exact values.

## 5 Conclusion

This paper presents DTIOD, a model for short-term metro outflow prediction that integrates long-term and short-term inflow dependencies. Unlike mainstream spatiotemporal fusion models that rely on explicit spatial modules, DTIOD employs a cross‑branch attention mechanism to implicitly learn inter‑station interactions, thereby enhancing robustness under limited spatial priors or incomplete data. In addition, sample-level OD matrices are incorporated as attention biases, providing a temporal granularity intermediate between global static and time-step-specific representations. This design enables the model to capture dynamic flow relationships while maintaining controlled computational cost.

Experiments on Hangzhou Metro AFC data demonstrate that DTIOD consistently outperforms mainstream spatiotemporal fusion models in prediction accuracy while retaining high training efficiency. Notably, these performance gains are achieved without predefined graph structures, external temporal labels, or auxiliary data, highlighting the model’s data efficiency and practical robustness. These advantages indicate strong potential for real-time short-term forecasting in urban rail systems, although performance at stations with highly volatile peak-hour flows remains a topic for further investigation.

Future work can be pursued in several directions. First, incorporating meteorological variables, temporal contextual information, and other heterogeneous data sources may further improve performance in environments where external conditions strongly influence travel demand. Second, extending the inflow–outflow dependency framework to related transportation forecasting tasks would help clarify its generality and applicability. Finally, integrating information from spatially adjacent alternative travel modes, including bike-sharing usage, public transport availability, and bus ridership, may further enhance short-term metro passenger flow prediction.

## Supporting information

S1 Fig Architecture of the DTIOD model. (JPG)

S2 Fig Dual-temporal cross-attention module for inflow–outflow dependency modeling. (JPG)

S3 Fig Pearson correlation between inflow and outflow sequences at four stations. (JPG)

S4 Fig Training dynamics of DTIOD and baselines. (JPG)

S5 Fig Prediction error comparison between DTIOD and baselines. (JPG)

S6 Fig Predicted vs. actual outflow at four representative stations. (JPG)
