# Supplementary figures and images for: Dual-temporal inflow–outflow dependency modeling for short-term metro outflow prediction
Source: PLoS One. 2026 Apr 21;21(4):e0347131. doi: 10.1371/journal.pone.0347131 (PMC13098924; doi:10.1371/journal.pone.0347131)

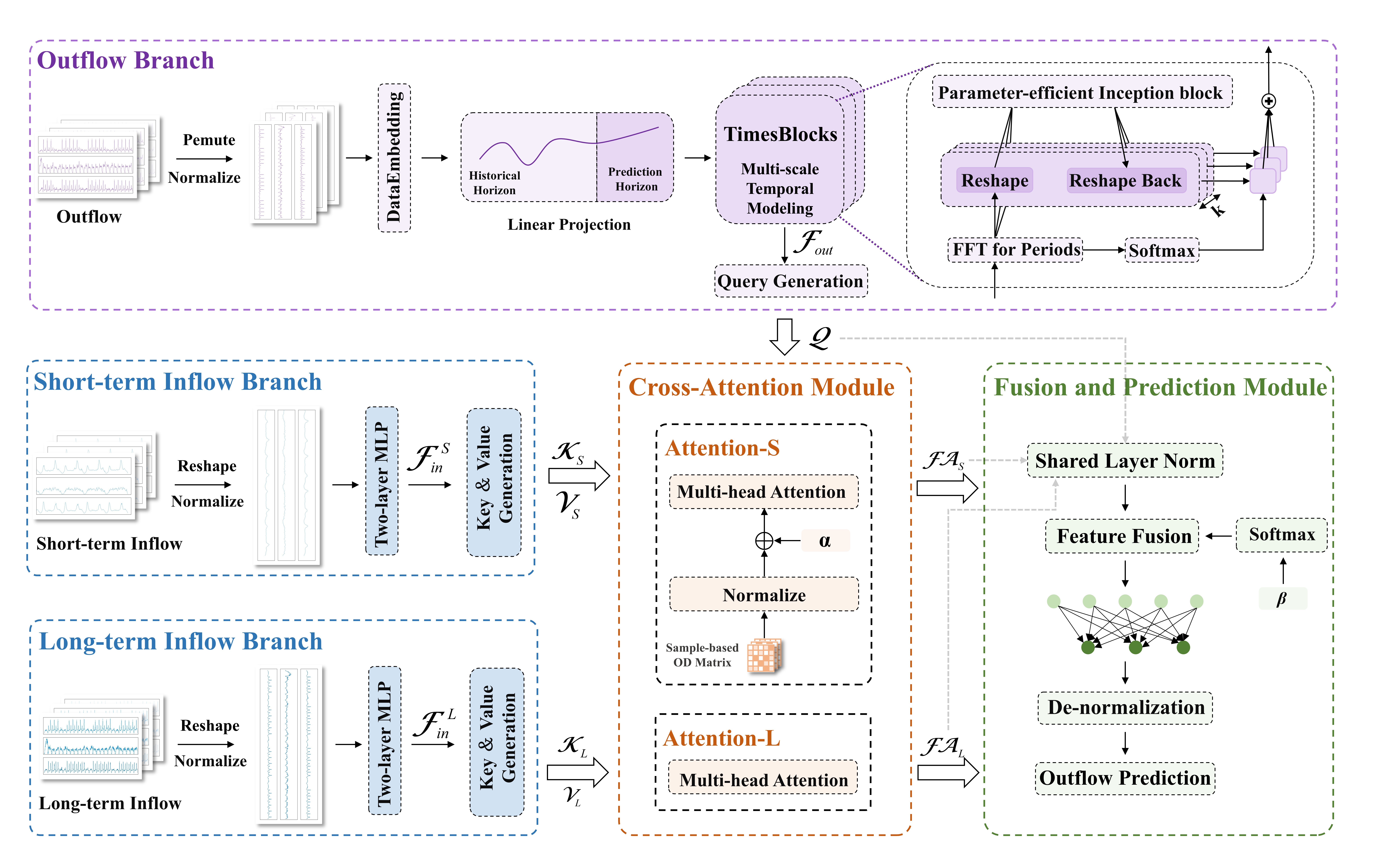

Supplement: S1 Fig — (JPG) [file pone.0347131.s001.jpg]

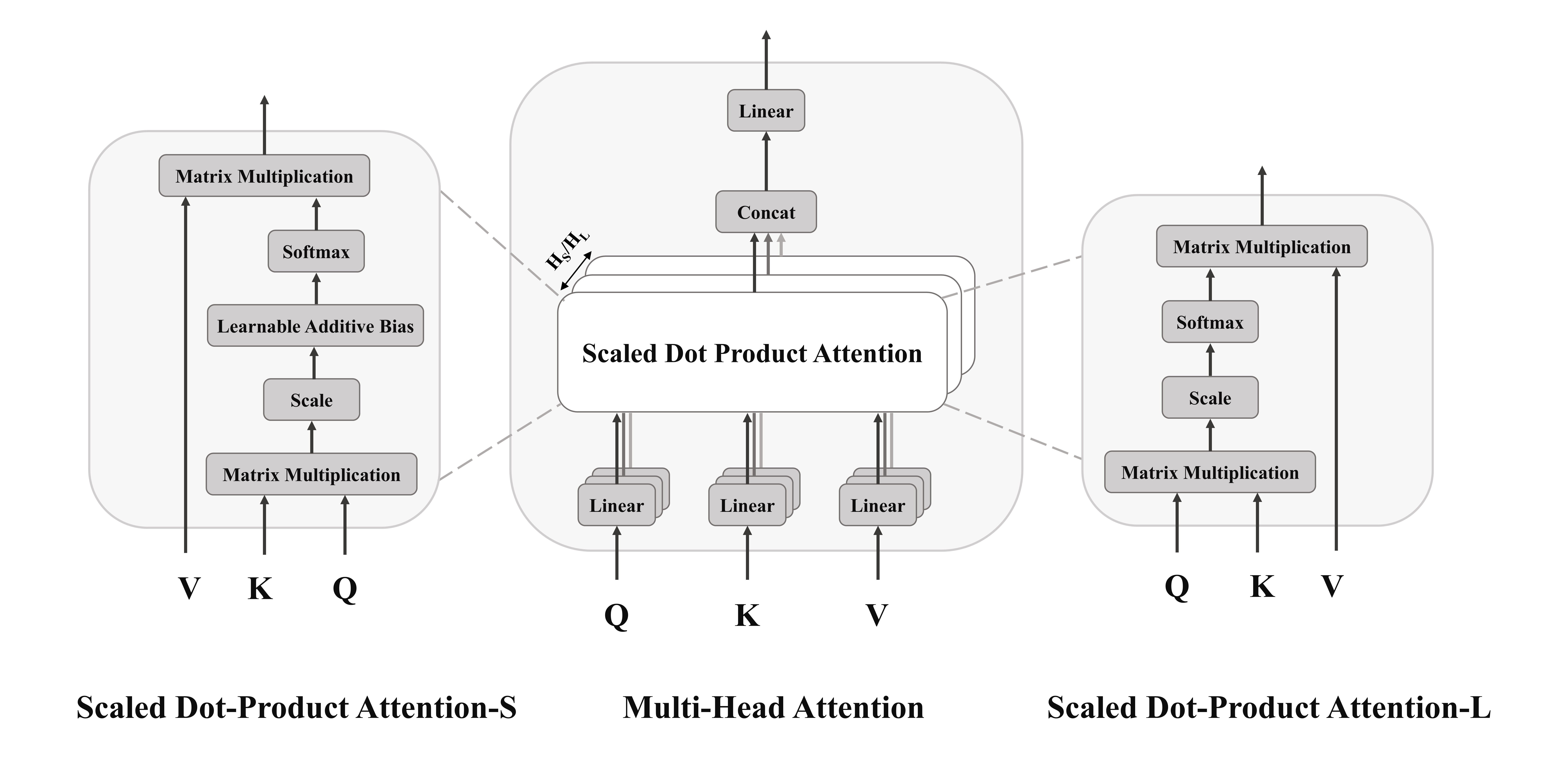

Supplement: S2 Fig — (JPG) [file pone.0347131.s002.jpg]

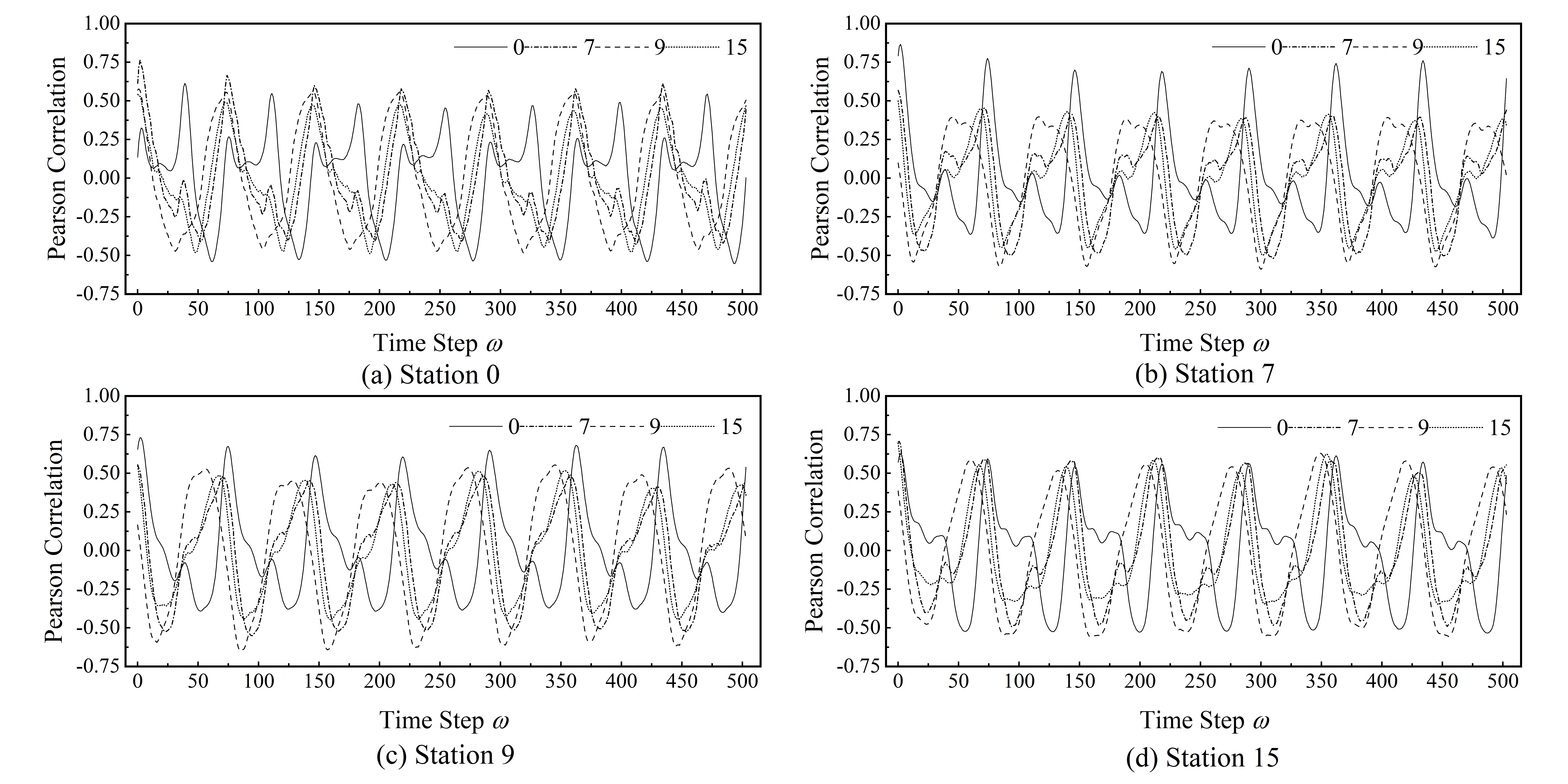

Supplement: S3 Fig — (JPG) [file pone.0347131.s003.jpg]

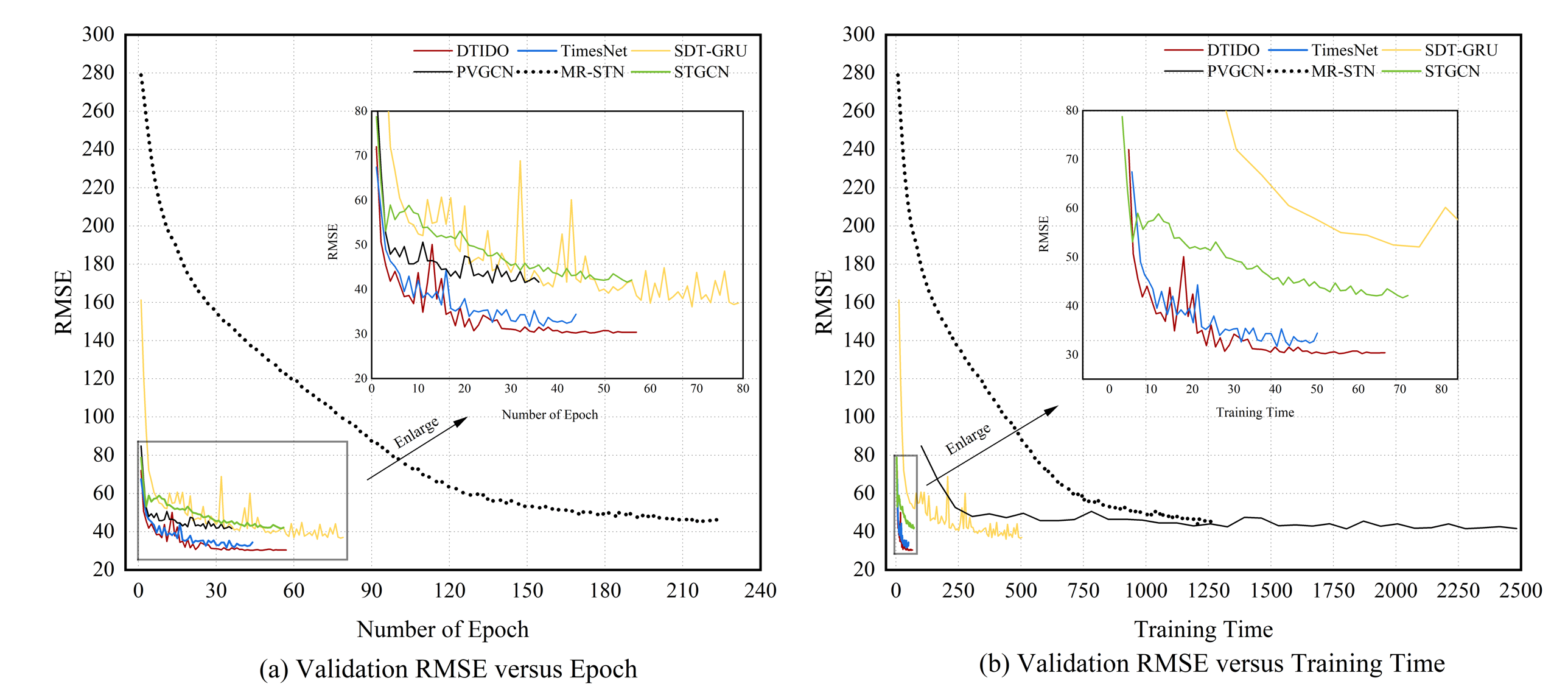

Supplement: S4 Fig — (JPG) [file pone.0347131.s004.jpg]

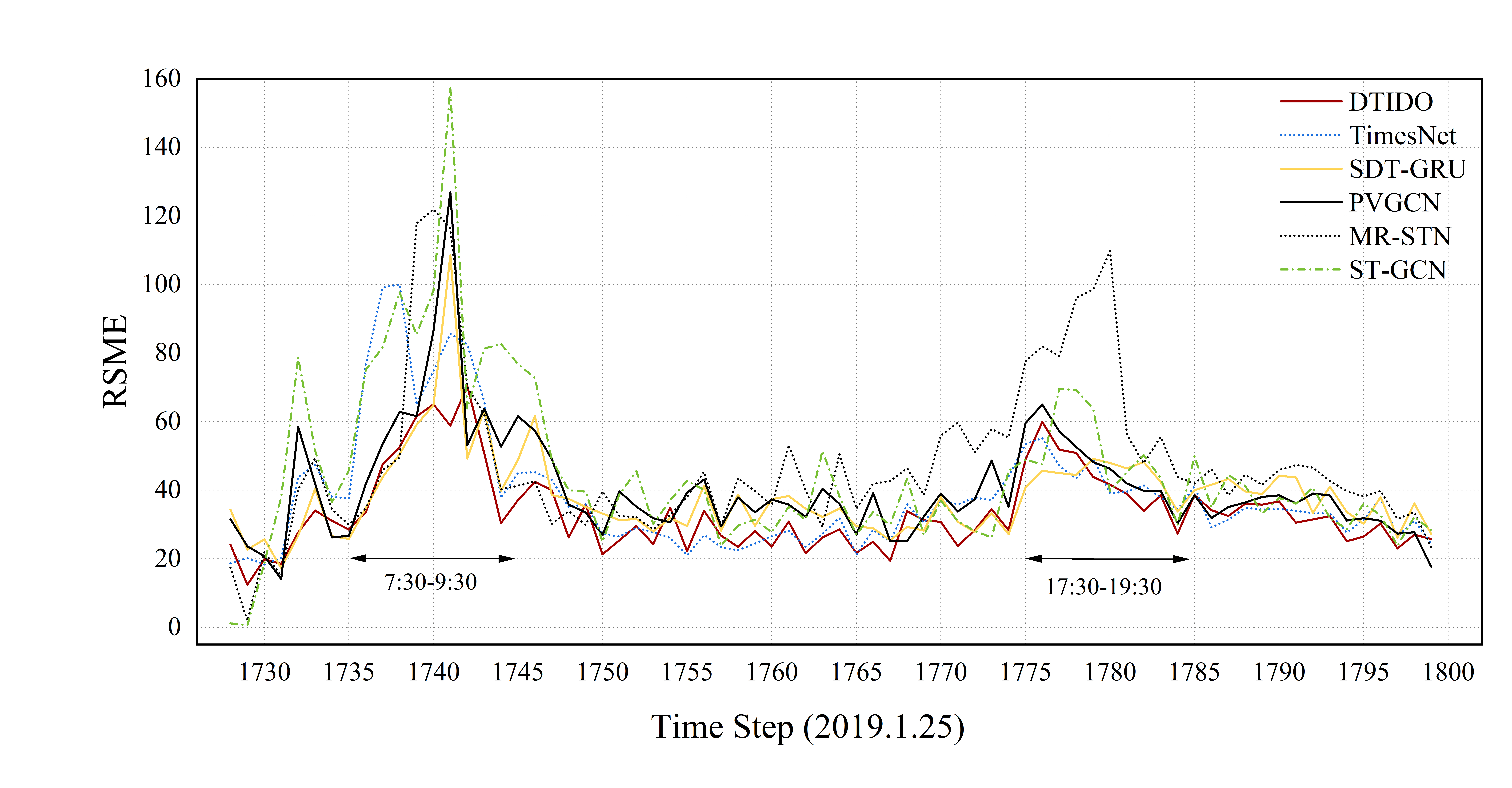

Supplement: S5 Fig — (JPG) [file pone.0347131.s005.jpg]

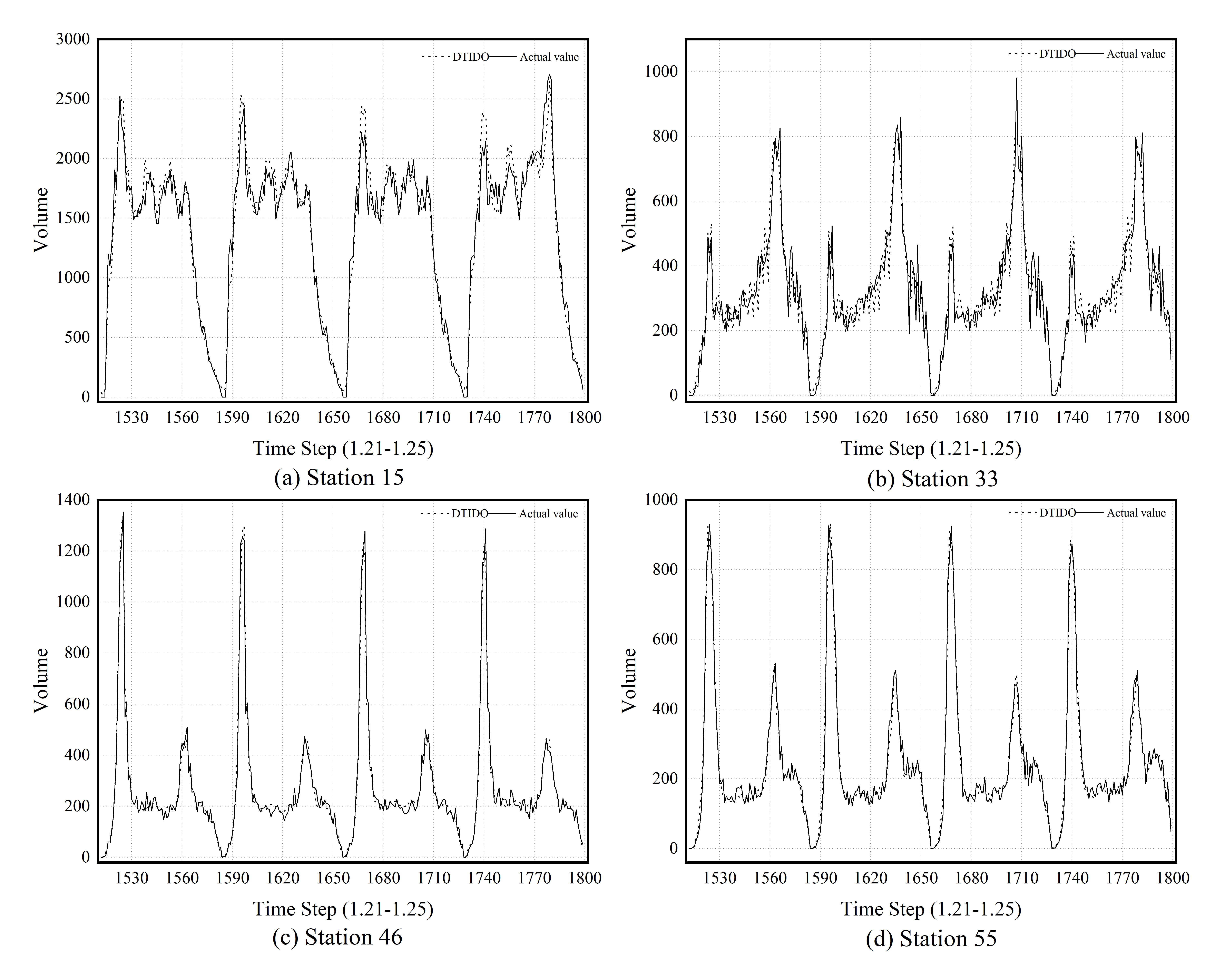

Supplement: S6 Fig — (JPG) [file pone.0347131.s006.jpg]
